# The Role of the Intrauterine Environment in Shaping Childhood and Adolescence Metabolic Outcomes

**DOI:** 10.3390/metabo15040252

**Published:** 2025-04-06

**Authors:** Asli Derya Kardelen, Feyza Darendeliler

**Affiliations:** 1 Division of Pediatric Endocrinology, Department of Pediatrics, Istanbul Faculty of Medicine, Istanbul University, Istanbul 34093, Türkiye; feyzad@istanbul.edu.tr; 2 Department of Genetics, Institute of Graduate Studies in Health Science, Istanbul University, Istanbul 34093, Türkiye

**Keywords:** childhood, diabetes, epigenetics, genetics, gestational, intrauterine environment, maternal, metabolic syndrome, obesity, preeclampsia

## Abstract

Emerging research suggests that the intrauterine environment plays a critical role in predisposing individuals to metabolic syndrome (MetS), a constellation of conditions that heightens the risk for heart disease, stroke, and diabetes. Traditionally linked to lifestyle, the risk for MetS is now understood to be also influenced by fetal exposures. The environment in which a child lives offers abundant potential sources that can contribute to an increased risk of developing various diseases, and in some cases, these factors can be avoided. This review integrates findings from both epidemiological and experimental research to underscore the impact of prenatal factors, including maternal nutrition, obesity, gestational diabetes (GDM), and birth size, on the subsequent development of metabolic derangements in offspring, particularly during puberty. The progression of genetic and epigenetic studies has enlightened the pathophysiology of these conditions starting in the intrauterine period and continuing into early life. By examining data and studies, this article elucidates the prenatal influences and underlying mechanisms that contribute to the pathogenesis of MetS. The updated understanding of the link between the intrauterine environment and future health comorbidities will draw attention to intrauterine care and maternal health and contribute to the prevention of serious diseases in adulthood.

## 1. Introduction

Metabolic syndrome (MetS) is a collection of cardiovascular risk factors and is associated with future chronic diseases in adulthood because of insulin resistance (IR), oxidative stress, systemic inflammation, and cellular dysfunction [1]. Although there is no precise definition of MetS in children and adolescents, the criteria used in adults have been adapted for youngsters. The components include hyperinsulinemia, arterial hypertension, dyslipidemia, and abdominal obesity. Abdominal obesity is the main component of MetS, and waist circumference measurement is the best indicator of abdominal obesity in clinical practice [2]. Excessive body weight is linked to a higher risk of cardiovascular disease (CVD), type 2 diabetes (T2DM), and MetS, particularly when fat accumulates in the abdominal area and when obesity starts in childhood [3]. Abdominal obesity is an important risk factor for systemic inflammation. In abdominal obesity, visceral fat deposits become enlarged and dysfunctional and produce inflammatory biomarkers like C-reactive protein (CRP), cytokines, tumor necrosis factor-alpha (TNF-a), prostaglandins, and leptin [4,5]. Because of the strong link between MetS and obesity, the resolution of MetS is achieved through the treatment of obesity [1].

According to the Global Burden of Disease Study, the occurrence of childhood obesity has doubled in more than 70 countries since 1980. The estimated number of children with obesity globally is 115.1 million, constituting a prevalence of 5% [6,7]. Longitudinal data indicate that children with obesity have a greater than 80% chance of continuing to be affected by obesity in adulthood [8].

MetS is frequently observed in conjunction with obesity, particularly in high-income countries where obesity rates are higher. The prevalence of MetS is increasing as well; however, its frequency shows diversity because of the different cut-off levels of MetS components [3,9]. Differences in the definitions of MetS also hinder the ability to draw comparisons across studies [10,11].

Some of the most commonly used MetS definitions are shown in Table 1 [12,13,14,15]. Tropeano et al. conducted a systematic review of the definitions of MetS in children and adolescents, concluding that the definition created by the International Diabetes Federation (IDF) is the most practical for clinical use [11,16] (Table 2).

Genetic and epigenetic factors, along with fetal and maternal factors, play an important role in the development of MetS [9]. There is evidence that an unfavorable intrauterine environment may predispose the development of MetS [9,17,18]. Fetal programming, rooted in the developmental origins of health and disease theory, describes the fetus’s adaptive responses to adverse environmental conditions during gestation. The environmental factors encountered by the fetus during pregnancy can have long-term effects on an individual’s physiology, metabolism, and overall health status. Adverse intrauterine environments, including insufficient nutrition, stress, hormonal disturbances, and toxin exposure, can influence fetal development and drive adaptive responses. However, these adaptations may heighten the risk of chronic diseases such as obesity, diabetes, cardiovascular disorders, and MetS in later life [19,20].

In the 1980s, David Barker first raised the issue of the early origins of adult diseases [17,18,20]. He observed an elevated risk of CVD, T2DM, and MetS in children born small for gestational age (SGA). This led to the creation of Barker’s hypothesis, in which alterations in fetal nutrition may result in developmental adaptation, causing cardiovascular and metabolic disorders in adulthood [17,18]. The Barker hypothesis was also supported by other studies [21]. A wide variety of factors have been investigated to elucidate these multivariate intrauterine factors underlying MetS.

In conclusion, based on all this evidence, although the underlying mechanisms and pathways have not yet been clarified, there is an association between the intrauterine environment and MetS.

## 2. Materials and Methods

In this review, we compiled studies that examine the intrauterine environment and its effects on future MetS. We started by providing a general introduction to and definition of MetS. We then examined the impact of intrauterine growth on the development of MetS, considering conditions such as intrauterine growth restriction (IUGR), SGA, and large for gestational age (LGA). Additionally, we explored the effects of maternal factors, including gestational diabetes mellitus (GDM), maternal obesity, nutritional status, vitamin deficiencies, maternal hypertension, preeclampsia, and epigenetic modifications on long-term metabolic consequences.

We conducted the literature search using the PubMed and Cochrane Library databases, focusing on studies published in English. Publications from other databases or studies written in languages other than English were not included. In our review, we prioritized systematic reviews, randomized controlled trials, and large cohort studies (such as national registries). Although we conducted a literature search covering all relevant subheadings, we did not use standard MeSH terms, which is a limitation of our review.

## 3. Impact of Intrauterine Growth on the Development of MetS

### 3.1. Intrauterine Growth Restriction (IUGR) and SGA

Intrauterine growth occurs in a balanced way that involves the integrity of maternal, placental, and fetal factors. Maternal factors include nutrition, chronic diseases, medication use, genetic factors, uterine anomalies, etc. Problems with any of these factors can affect fetal growth [22]. There are three possible outcomes of intrauterine growth on birth, and these are appropriate for gestational age (AGA), large for gestational age (LGA), and SGA. SGA is defined as birth weight and/or height below −2 SDS for mean gestational age [22,23]. Although intrauterine growth restriction (IUGR) and SGA are often used interchangeably, they have different definitions, and over time, both can lead to overlapping outcomes with similar mechanisms [22,23]. The diagnosis of IUGR is based on antenatal ultrasonography (USG) follow-up, and neonates with IUGR may have normal weight at birth. Children that are born AGA with fetal growth restriction may have similar long-term problems as children that are born SGA, but it would be difficult to distinguish between these children when they are born AGA [24]. The conditions that lead to reduced fetal growth are classified into fetal, placental, maternal, and environmental factors. These factors alone or interacting together can cause SGA birth [24,25]. Maternal conditions such as hypertension, preeclampsia, infections, hypothyroidism, and malnutrition can result in SGA births [25]. One of the most fundamental things to prevent SGA birth is to ensure the mother’s proper nutrition [25].

In observational studies, both high and low birth weights are associated with an increased risk of MetS [26]. According to Barker’s hypothesis, low birth weight creates a predisposition to future T2DM and CVD in early adulthood [27]. There have been other studies supporting this hypothesis ‘developmental origins of health and disease’. While the exact mechanism remains unknown, it is proposed that certain predisposing factors may be responsible for this condition, one of which is early catch-up growth.

After birth, postnatal 3 and 6 months is the period of the fastest growth, and catch-up growth is most often in weight rather than height [28]. The rapid catch-up in birth weight, particularly in SGA children, is the major determinant of future metabolic problems like obesity and CVD [29,30,31]. In healthy catch-up, growth should be equal in weight, height, and lean body mass [32]. SGA-born children have a lower amount of fatty tissue. This reduced amount of adipose tissue consists of mainly visceral fat rather than subcutaneous adipose tissue, resulting in an increased ratio of visceral-to-subcutaneous fat [29,33]. During catch-up growth, the rise in fat mass occurs earlier and to a larger extent compared to the increase in muscle mass [34].

In the Avon longitudinal study of pregnancy and childhood (ALSPAC), IUGR children who had catch-up growth (greater than 0.67 SD) in the first two years of life were taller and heavier [35]. They had higher body mass index (BMI), higher body fat, and larger waist circumference at 5 years of age compared to their peers [36]. Further studies with the same cohort of children who had higher weight gain in the first three years showed decreased insulin sensitivity, higher BMI, and increased waist circumference [37]. In another study, AGA and SGA children born prematurely did not show IR compared to full-term children, as long as they made catch-up growth to attain their target height and maintain a normal BMI [38]. In this study, basal insulin levels were found to be similar in preterm AGA and term AGA children with similar and normal BMI levels [38]. In preterm SGA children, insulin levels were similar to those in preterm AGA children but were significantly lower compared to term SGA children [38]. HOMA-IR (Homeostatic Model Assessment for Insulin Resistance) yielded similar results. It appears that SGA-born children are more likely to experience future health complications compared to preterm children with normal BMI, especially in early childhood [38].

Adipose tissue secretes various adipokines into the bloodstream, leading to decreased insulin sensitivity in peripheral organs. These molecules include adipomyokines, such as serum interleukin-6 (IL-6), TNF-a, visfatin, myostatin, irisin, and glypican-4, and hormones, such as leptin and resistin [9]. Identifying the relationship between the intrauterine environment and various pathways regulated by these molecules poses a significant challenge.

Adiponectin, a peptide with anti-inflammatory properties, plays a crucial role in determining insulin sensitivity. Low levels of adiponectin have been linked to IR in both animal and human studies [39]. Few studies have investigated adiponectin levels in prepubertal children born SGA, but the results have been inconsistent, showing low, normal, or even high levels despite hyperinsulinemia [39,40,41,42,43,44]. 

In a previous study with preterm SGA and AGA children, leptin, adiponectin, visfatin, insulin levels, and HOMA-IR did not show a significant difference between the groups [45]. In another study, infants that were born SGA with a BMI in the higher range of the sample distribution, albeit still within normal limits, showed lower levels of adiponectin when compared to AGA controls [46].

IGFBP-1 has been proposed as another potential marker for IR in children with obesity [47]. In one of our studies, we found no statistically significant difference between IGFBP1 levels in SGA children without obesity compared to AGA controls [46]. Nevertheless, the levels of IGFBP-1 in SGA children have shown variability in various studies, with some indicating a decrease [48] and others showing no significant difference [49] compared to children with a normal birth weight.

Ghrelin, functioning as a natural ligand for the growth hormone (GH) secretagogue receptor, may have a key role in controlling growth and energy metabolism equilibrium from fetal life to adulthood [50]. Ghrelin has an inverse relationship with BMI, and it increases in anorexia and cachexia and decreases in obesity [51,52]. Insulin levels have a strong negative correlation with ghrelin levels [51,52]. In a study by Gohlke et al., ghrelin was found to be a prognostic factor for postnatal catch-up growth in SGA children, and those with high ghrelin levels exhibited better catch-up growth than children with low ghrelin levels [53]. In another study, ghrelin levels were higher in preterm children, regardless of whether they were born SGA or AGA, when compared to term children in prepubertal stages [54]. Ghrelin levels are lower in term SGA children than in preterm SGA children at prepubertal ages, and this may have an association with elevated insulin levels in this group [54]. However, a recent review suggests that the role of ghrelin in SGA children and its impacts on metabolism should not be limited to ghrelin levels alone [49].

According to Rabinowicz et al. [55], preterm SGA infants exhibited elevated triglyceride levels in comparison to their age-matched AGA counterparts. A recent meta-analysis demonstrated that rapid growth was inversely related to HDL, with a weighted mean difference of −0.068 (95% CI: [−0.117, −0.020]). However, no significant associations were found between rapid growth and levels of triglycerides, total cholesterol, or low-density lipoprotein cholesterol [56]. Research has indicated that low birth weight is associated with impaired endothelial function during both childhood and adulthood [57,58]. In SGA children, increased carotid intima-media thickness, which is associated with atherosclerotic vascular changes and serves as an indicator of cardiovascular risk, can be observed as early as 3–6 years of age [59]. Another little-known effect of low birth weight is the emergence of hypertension and chronic kidney disease, which is attributed to low nephron numbers. Preterm birth or low birth weight (LBW) may cause a low nephron number that creates an increased risk of proteinuria, chronic kidney disease, and hypertension later in life [60,61], but there is controversy on this issue. In a study conducted with children without obesity who were born SGA at term and AGA controls, there was no association between blood pressure and current BMI, birth weight, and birth height in regression analysis [62]. Creatinine, glomerular filtration rate (GFR), urea, and microalbumin/creatinine levels between AGA and SGA children did not show significant differences [62]. The results of this study suggested that there is no apparent effect of IUGR on blood pressure in childhood, and there is no evidence to suggest a negative correlation between birth weight and blood pressure in children [62]. Contradictorily, another study showed an increased prevalence of kidney dysfunction in children with LBW in contrast to those born with a normal birth weight by using four GFR estimating formulas and examining 6336 National Health and Nutrition Examination Survey (NHANES) participants, a sample reflecting more than 13 million children in the US [63].

In conclusion, additional research is needed to determine the factors that contribute to metabolic problems in people born with SGA. In general, the presence of risk factors like overweight, obesity, ethnicity, and family history can increase the risk of metabolic disorders. Routine monitoring of metabolic parameters is not recommended for all SGA-born children but is suggested for those with one or more risk factors [22].

### 3.2. LGA

LGA is defined as birth weight and/or height above +2 SDS for mean gestational age [64]. The underlying causes of LGA birth are multifactorial and include maternal obesity, gestational diabetes mellitus (GDM), rapid weight gain during pregnancy, and maternal hyperlipidemia [65]. It is important to highlight the link between maternal GDM and overgrowth, as the sustained exposure to high glucose levels can trigger the fetus to overproduce IGF-1 and insulin, resulting in macrosomia [66].

Epidemiological studies indicate a U-shaped relationship between birth size and long-term health outcomes, and they show that both SGA and LGA babies are at higher risk for the development of CVD [67]. Meta-analyses have shown that children born LGA have a 1.5–1.7-fold increased risk of being obese or overweight in adulthood [68,69]. Another systematic review supported the relationship between being born LGA and childhood hypertension [70]. In a longitudinal study, children born over the 85th percentile of weight were more prone to being overweight at ages 6 years (OR 1.8), 9 years (OR 2.1), and 15 years (OR 2.0) in comparison to their counterparts [71]. However, there are also studies showing that there is no relationship between birth weight and obesity in adulthood [72]. Kampmann et al. [73] did not find a U-shaped association between birth size and future cardiometabolic risks. According to their results, body size during childhood and adolescence was a reflection of birth size. Nevertheless, they did not find potential adverse metabolic effects between the ages of 9 and 16 that emerged from being born SGA or LGA.

Children that are born LGA are rich in both fat mass and lean body mass [74]. LGA may represent fetal obesity, and fetal obesity can be transmitted from childhood to adulthood. However, more than half of the children born LGA can make catch-down growth after birth [74]. Bueno et al. found that LGA infants who experienced catch-down growth had a lower BMI (25 ± 4 vs. 29.4 ± 4 kg/m^2^ *p* = 0.0009) and abdominal circumference (84.7 ± 13.2 vs. 93.9 ± 10.7 cm *p* = 0.009) at 25 years compared to those who had overgrowth [75], but the future impact of catch-down growth is not clearly known yet.

**Figure 1:**
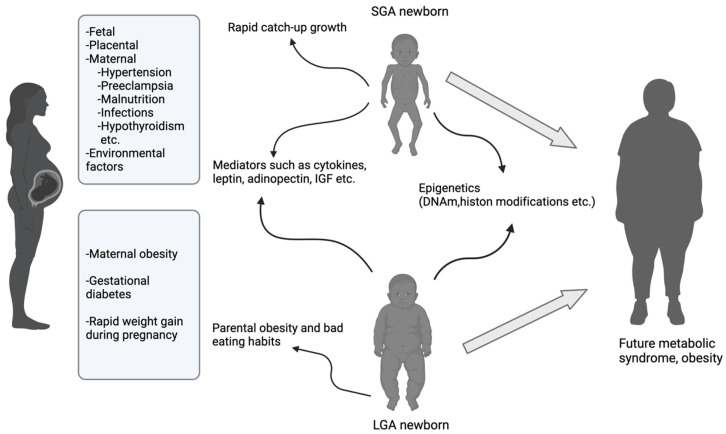
The relationship between SGA and LGA birth with metabolic syndrome (created with BioRender (licensed version)).

Several hypotheses and mediators have been suggested to explain why children born with a high birth weight are more likely to develop MetS later in life. One of these is parental obesity [76]. Children born LGA often have parents with obesity. Parents can predispose future obesity and MetS of their LGA children through their dietary and exercise habits. Parents can pass on their genetic predispositions to children and can cause susceptibility to future obesity [76]. Figure 1 depicts the relationship between SGA and LGA and the subsequent development of MetS later in life.

Mediators like adipokines, cytokines, and IGF levels were investigated to understand the underlying mechanisms with no clear conclusion. Challa et al. [77] investigated 98 children without obesity between 5.5 and 8 years of age and divided them into two groups, LGA and AGA. IGF-1, free IGF-1, and IGFBP-1 levels did not differ between groups. They found that prepubertal children with LGA had increased IR indices and leptin levels, which may have implications for future metabolic characteristics [77]. In another cross-sectional study, prepubertal children born with LGA were compared in terms of IL-6, TNF-a, leptin, IGF-1, and IGFBP-1 concentrations and their relationship with IR [78]. Their findings indicated that LGA-born children had higher fasting insulin, HOMA-IR, IL-6, and leptin levels and lower TNF-a levels compared to AGA-born children [78]. Regression analysis revealed that HOMA-IR was best explained by (R2 = 0.517) birth weight SDS (ß = +0.418, *p* = 0.002), leptin (ß = +0.620, *p* = 0.000), and TNF-a (ß = −0.374, *p* = 0.003) in LGA-born children [77]. In another study, IGFBP-1 and leptin levels did not show a difference between LGA and AGA children [79]. However, adiponectin was significantly lower in the LGA group, indicating a potential for future IR within this group [79]. In summary, further research is necessary to better understand the potential link between these mediators and future MetS development in LGA-born children.

Another suggested problem is the predisposition of LGA babies to type 1 diabetes (T1DM). According to the results of a recent meta-analysis, children that were born LGA had higher odds of developing T1DM (OR = 1.28, 95% CI: 1.15–1.43), obesity and overweight (OR = 1.44, 95% CI: 1.31–1.59), MetS (OR = 1.43, 95%; CI: 1.05–1.96), and hypertension (OR = 1.23, 95% CI: 1.01–1.51) in the future compared to children born AGA [80]. The association of LGA with T1DM was unexpected. Stene et al. [81] demonstrated a linear relationship between increased birth weight and T1DM after adjusting potential influencing factors such as GDM (95% CI 1.24 to 3.94, *p* = 0.0001). Fetal overgrowth is thought to cause immune-mediated β-cell destruction, but current evidence is not enough to support this.

## 4. Impact of Maternal Obesity and Gestational Diabetes Mellitus on the Development of MetS

The incidence of maternal obesity, a major public health problem, has been increasing in recent years and is observed in approximately 35% of women of reproductive age [82]. Pregnant women with obesity are naturally prone to pregnancy complications such as GDM, thromboembolism, preeclampsia, and congenital abnormalities of the fetus [83]. The likelihood of being born LGA is increased in babies of mothers with obesity. Obesity and GDM may cause increased mortality and morbidity in both the mother and offspring [83]. Both conditions are associated with chronic, low-grade inflammation, namely “metainflammation”, which is possibly responsible for future adult disorders of the offspring [84]. Epidemiological studies demonstrated that maternal obesity has a relationship with obesity [85], CVD [86], and T2DM [87] of the child later in life. Studies have also been conducted on how maternal weight control influences the child’s health, and they have indicated different results [88,89]. Gestational weight gain restriction for pregnant women with obesity (BMI > 30 kg/m^2^) was found to have a positive impact on the child’s weight at five years of age when compared with a sibling born without intervention [88]. A different study showed that women who had bariatric surgery had better pregnancy outcomes, but there was no long-term decrease in the risk of obesity for their children born before or after the surgery when they reached preschool age [89]. They attributed this result to the bad eating habits of the families.

Gestational diabetes, which occurs in the second or third trimester of pregnancy, is one of the most common complications of pregnancy [90]. Insulin and glucose levels are elevated in women with GDM as a result of decreased insulin sensitivity. In GDM, glucose passes through the placenta while insulin does not, leading to high blood glucose levels and insulin production in the fetus, likely contributing to fetal overgrowth [91,92]. Additionally, pregnant women receiving insulin showed increased fetal growth rates in comparison to those with normal glucose tolerance and those with GDM managed through diet [93].

Exposure to high maternal glucose levels in utero is thought to cause an increased risk of MetS in later years. During fetal development, insulin promotes abdominal growth, and abdominal circumference reflects the size of the liver [94] and abdominal adiposity, which is linked to a higher weight-for-height z score in young children [95]. Apart from glucose, high levels of fatty acids and amino acids that are potentially influenced more by elevated BMI rather than GDM may also have an impact [93,95]. In a previous study, GDM-affected pregnancies exhibited increased fetal growth and a higher risk of offspring being overweight between ages 5 and 9 years [93]. The growth rate of the fetus in the initial part of the third trimester acted as a mediator for up to 15% of this relationship, while the pre-pregnancy BMI had a strong effect [93].

In a systematic review, a marked rise in the risk of MetS in children whose mothers had GDM during pregnancy was detected (RR 2.07, 95% CI 1.26–3.42) [96]. Although observational epidemiological studies support this conclusion [90], there are also some debates on this subject. According to some studies, this effect may not be a direct effect of GDM but of the associated LGA birth, which is known to be a cause of obesity in youth [97], and it is still uncertain if elevated blood glucose levels below the GDM threshold are linked to an increased risk of MetS during childhood [98]. Recently, Bendor et al. [99] provided new evidence that mothers with high glucose levels but no diabetes also had children with obesity later in life. According to their results, this association seemed to be more pronounced with the severity of maternal obesity [99].

During gestation, there are critical periods in which the fetus is actively shaped by the internal and external stimuli. Exposure to high-fat diets dysregulates the expression of key genes involved in oxidative stress, fetal growth, and hepatic gluconeogenesis [100,101].

Moreover, hypothalamic blood flow in children exposed to GDM by ≤ 26 weeks of gestation was found to be increased when stimulated by glucose compared to children nonexposed to GDM, and these results persisted even after controlling variables such as the age of child, sex, BMI, and maternal BMI [102]. Additionally, heightened hypothalamic response to glucose was a predictor of increased adiposity in the child after one year, and these hypothalamic changes have been implicated in future risk for obesity [102].

In recent years, there has been growing interest in the role of inflammation in programming metabolic disorders in individuals exposed to adverse intrauterine conditions due to maternal obesity. Metainflammation is not the same as an acute pro-inflammatory reaction and is primarily initiated by metabolites and nutrients, leading to systemic IR [103]. In particular, TNF-α, IL-6, and CRP are reported to be significantly high in the circulation of pregnant women with obesity or GDM [84].

An increase in TNF-α levels in pregnancy, which mainly originates from the placenta, correlates with IR independent of adipose tissue. TNF-α impairs insulin signaling, and IR in pregnancy is attributed to this cytokine [104]. There is evidence that maternal obesity causes adipogenesis and IR in fetal muscle, leading to future obesity and diabetes in the offspring. The mechanism of enhanced adipogenic differentiation in fetal muscle may be due to oxidative stress and inflammatory response [101]. Oxidative stress due to high glucose levels induces adipogenesis in fetal muscle, creating a milieu that predisposes obesity.

In animal models, the decreased AMP-activated protein kinase activity of fetal muscle, possibly due to increased TNF-α levels, may explain the IR in GDM mothers [105]. Skeletal muscle makes up 40–50% of the whole body and is one of the main places where glucose and fatty acids are used. Skeletal muscle is one of the main sites of IR in individuals with obesity. Adipogenesis and myogenesis are two competing processes; the inhibition of one process promotes the other process. Both cell types, adipocytes and myocytes, originate from mesenchymal stem cells [106,107]. In animal models, maternal obesity has been shown to trigger an inflammatory response in fetal muscle and cause IR and adipogenesis in muscle cells due to the upregulation of Toll-Like receptor 4/Nuclear Factor-κB signaling [105]. However, the effect of this increased inflammatory response on the fetus may be variable. Studies have yielded different results. Ategbo et al. found that leptin, TNF-α, IL-6, and IL-10 levels were decreased in macrosomic infants compared to their GDM mothers [108]. The placenta is the main source of cytokines required for the initiation and maintenance of pregnancy and plays a pivotal role in shaping the fetal environment. It was found that placental macrophages increased 2–3 times in mothers who have obesity, with an increase in cytokines such as IL-1, IL-6, TNF-α, and mRNA and protein expression [109,110,111,112]. However, this increased inflammatory response may not be transmitted in the fetus in the same way [84]. This brings the question ‘does the placenta act as a buffer protecting the baby from the excess inflammatory response in this structure?’. The placenta is the source of inflammation; however, it limits the transfer of the inflammation to the fetus and acts as an adaptive mechanism [104].

In conclusion, metainflammation seems to affect the fetus, but the role of the placenta and long-term implications remain unclear, with the possibility of other contributing factors.

## 5. Maternal Nutrition

Adequate intake of vitamins and micronutrients is essential for maintaining the body’s normal functioning, growth, and development. Micronutrient deficiencies, characterized by insufficient essential vitamins and minerals, are particularly concerning during pregnancy due to increased nutritional demands for both the mother and fetus. These deficiencies can contribute to adverse maternal and fetal outcomes, including preterm birth and low birth weight [113]. Studies have reported that maternal nutritional restriction may lead to metabolic diseases in adulthood [114,115]. A fetus experiencing inadequate nutrition in the womb adapts by developing a more efficient metabolic profile to enhance survival. This process is linked to IR, impaired pancreatic beta-cell function, and altered fat storage mechanisms [116].

Epidemiological studies in humans have demonstrated a link between early life episodes of nutritional restriction and the later development of MetS-related conditions, including obesity and T2DM. In these cases, an increase in glucose transporter 4 (GLUT)-mediated glucose uptake in skeletal muscle and enhanced lipogenesis in adipose tissue has been reported [117,118]. Another proposed mechanism suggests that nutritional elements, including vitamins, trace elements, carbohydrates, amino acids, and lipids, influence gene activity and expression in the uterus and during early life development by modifying epigenetic processes. These alterations may increase the child’s susceptibility to developing MetS later in life [119,120].

A study analyzing cord blood to assess oxidative stress levels in SGA infants born to undernourished mothers compared to AGA infants born to healthy mothers found increased oxidative stress in the SGA group. This was indicated by higher levels of malondialdehyde, a key product of lipid peroxidation, along with lower levels of the antioxidant glutathione and reduced activity of the antioxidant enzymes superoxide dismutase (SOD) and catalase compared to AGA controls [121]. Oxidative stress may be due to maternal vitamin deficiencies. Neonatal levels of vitamins A and E depend on maternal levels, and preterm infants have lower concentrations of lipid-soluble antioxidant vitamins in their serum compared to term infants, making them more vulnerable to oxidative stress [122]. Maternal 25-hydroxyvitamin D (25(OH)D) deficiency has been linked to metabolic alterations, a higher risk of chronic diseases, and increased adiposity in adulthood. In an animal model, researchers found that maternal 25(OH)D deficiency increases oxidative stress, contributing to the development of MetS in both the mother and offspring. This study found that 25(OH)D-deficient rats had lower Nrf2 and CBR1 levels, higher ROS levels, and reduced SOD levels in the placenta, liver, and pancreas, indicating impaired antioxidative processes. Offsprings had high triglycerides, FPG, insulin, and HOMA-IR levels, along with reduced HDL-c levels in peripheral blood. The authors also found that levels of inflammatory markers IL-1β, IL-6, and TNF-α were higher in the liver and pancreas of the offspring compared to the control group. According to the results, treatment with vitamin D may play a role in preventing MetS in the offspring [123]. A study conducted with six-year-old children found no association between maternal and cord blood 25(OH)D concentrations and cardiovascular risk factors in childhood. However, severe maternal 25(OH)D deficiency (<25.0 nmol/L) during pregnancy was linked to an unfavorable childhood body composition profile [124]. However, as randomized controlled trials have not demonstrated significant beneficial effects of vitamin D supplementation on diabetes incidence, glucose regulation, or IR in the general population, these findings remain inconsistent [125].

Vitamin B12 and folate serve as methyl donors in one-carbon metabolisms, playing a crucial role in DNA synthesis and epigenetic regulation, which in turn influence cell growth and differentiation. Consequently, they are key regulators of fetal development [126]. Additionally, low maternal vitamin B12 levels were linked to reduced B12 concentrations in offspring, both in cord blood and throughout childhood. This deficiency was also associated with IR and a higher risk of diabetes in childhood [127]. Furthermore, maternal vitamin B12 deficiency (<150 pmol/l) and high folate levels are linked to IR in offspring at 6 years of age. Lower maternal vitamin B12 levels at 18 weeks of gestation (*p* = 0.03) were associated with higher HOMA-IR in offspring [128].

Observational studies indicate that low maternal vitamin B12 levels, elevated homocysteine, or an imbalance between B12 and folate are associated with an increased risk of pregnancy complications, including recurrent pregnancy loss, GDM, and pre-eclampsia. Additionally, these factors have been linked to SGA births, IUGR, and adverse long-term health outcomes in offspring, such as increased adiposity and IR [127,129].

The etiology of MetS is multifactorial, involving not only maternal nutrition but also a variety of other contributing factors. Although much remains unknown, ensuring optimal maternal nutrition and addressing vitamin deficiencies may have a protective effect on potential metabolic outcomes in the offspring.

## 6. Maternal Hypertension and Preeclampsia

Hypertensive disorders during pregnancy are a major contributor to maternal and perinatal complications globally and are recognized as a potential factor in increasing the long-term risk of cardiovascular diseases in offspring. A previous systematic review indicates that pregnancy-induced hypertension is commonly associated with higher blood pressure in offspring. In utero exposure to preeclampsia was associated with an increase in systolic blood pressure of 2.39 mmHg, diastolic blood pressure of 1.35 mmHg, and a BMI of 0.62 kg/m^2^ in childhood [130]. Maternal hypertension may influence the development of fetal organs and vascular structures, potentially predisposing the child to adverse cardiometabolic health outcomes. The elevated blood pressure observed in offspring exposed to maternal hypertension may be programmed through intrauterine mechanisms. Although the mechanism remains unclear, it has been proposed that maternal and fetal glucocorticoids may play a role in this process [131]. Furthermore, high blood pressure is associated with adverse perinatal outcomes, including SGA and preterm birth, which may, in turn, contribute to metabolic consequences related to birth weight [132]. Moreover, the underlying cause of maternal hypertension is also a crucial factor in this context. If hypertension arises from maternal obesity or lifestyle factors, then the observed changes in the offspring may be attributed not only to hypertension itself but also to these coexisting maternal factors [133]. Preeclampsia leads to increased cardiac afterload in the fetus due to higher resistance in the placental blood vessels. This condition accelerates the maturation of cardiomyocytes, resulting in early asymptomatic alterations in fetal cardiac structure and function [134,135]. Microvascular adaptations [136] and endothelial dysfunction [137] have been identified in the offspring of mothers with preeclampsia. The imbalance in maternal vasoactive elements observed in preeclampsia or hypertension, characterized by elevated levels of vasoconstrictors (such as thromboxane A2 and endothelin) and reduced levels of vasodilators (such as prostacyclin and nitric oxide), may contribute to fetal vascular complications [138]. However, current evidence does not support a clear link between prenatal exposure to preeclampsia and childhood blood pressure. Additionally, no significant associations have been found between hypertensive disorders of pregnancy and childhood levels of cholesterol or glucose [139]. This systematic review suggests that preeclampsia or hypertension alone does not directly contribute to metabolic syndrome in childhood but may increase susceptibility when combined with genetic predisposition or lifestyle factors. Another meta-analysis conducted by Bi et al. confirmed the lack of association between in-utero exposure to preeclampsia and adverse lipid or glucose metabolism outcomes in offspring under 15 years of age (RR 1.07, 95% CI 0.88–1.32) [140]. Yet, this review is limited to children; the authors noted that the conclusions could be different if adults were also analyzed.

## 7. Impact of Epigenetic Modifications on the Development of MetS

The epigenome is a significant factor in bridging the gap in our knowledge of how early life events impact obesity and cardiometabolic disease. During the period from conception to the second year of life, epigenetic processes are most active [141]. Changes in epigenetic patterns in response to environmental factors, such as maternal and neonatal diet, can influence the risk of chronic diseases later in life [142]. Epigenetics involve mechanisms that cause heritable changes in gene expression without altering the DNA sequence, using methods such as DNA methylation (DNAm), noncoding RNA expression, and histone modifications.

DNA methylation has a key role in gene expression and can promote gene expression. In a large Norwegian study population, both positive and negative correlations were found between birthweight and cord blood DNAm in the *ARID5b* and *XRCC3* genes during a comprehensive epigenome-wide analysis. The down-regulation of these genes has been shown to increase leptin levels [143]. In another study, the analysis of DNA methylation across the genome in the IUGR group revealed a widespread trend towards increased methylation in the regulatory regions of CD3+ T cells, which are crucial for regulating adipose inflammation and insulin sensitivity [144]. The overexpression of specific micro-RNAs in dried blood samples from newborns with macrosomia has been observed, particularly those that target genes and pathways involved with FoxO and PI3K/Akt signaling and that are associated with cardiometabolic disease [145]. Another study aimed to analyze the differences in DNA methylation patterns in the placenta of infants that were born LGA revealed elevated levels of *CACNA1G* gene expression and reduced levels of *FKBP11*, *DECR1*, and *GNAS* genes compared to AGA samples, which correlated with DNA methylation levels of these genes [146]. Their findings of epigenetic variation and gene expression differences in LGA infants indicated that the intrauterine environment could lead to changes in the placental genome’s epigenetics and gene expression, potentially affecting abnormal intrauterine growth and future health outcomes. Moreover, GDM also has the potential to induce epigenetic abnormalities in the placenta, such as alterations in DNAm and miRNA expression, ultimately influencing the growth of the fetus [147,148]. A different study suggested that a high blood glucose environment in the womb changes the expression of GLUT, leading to increased glucose transfer from mother to fetus and resulting in higher offspring weight [149].

The involvement of epigenetic modifications in fetal developmental programming is evident. To gain a deeper insight into how interactions between the epigenome and the environment influence future health, it is imperative to develop novel analytic methods. These methods should concentrate on standardizing, annotating, and harmonizing epigenetic data to facilitate data integration [150].

## 8. Conclusions

Based on the reviewed evidence, substantial epidemiological data have shown that SGA birth, LGA birth, maternal obesity and nutrient deficiencies, GDM, and preeclampsia are risk factors for the development of MetS later in life.

Birth weight is often seen as a reflection of the prenatal environment and is linked to body fat levels throughout a person’s life, and there is evidence that, besides birth weight, catch-up growth has an impact on future health. SGA infants experiencing rapid catch-up growth may face negative consequences, whereas LGA infants undergoing catch-down growth may be advantageous in terms of future adiposity and cardiometabolic risk. Apart from growth rate, environmental, genetic, and epigenetic reasons, along with inflammatory markers or adipokines affected by the intrauterine environment, play a role in determining future metabolic outcomes. The occurrence of MetS is highly complex and can be attributed to all these reasons we mentioned, including the dysregulation of various metabolic pathways, most of them poorly understood, and their interaction with each other. Therefore, it is difficult to distinguish the effects of the intrauterine environment from postnatal factors. Today, even if we know that the intrauterine environment has an impact on later MetS, it is certain that very long and hard studies are needed to unravel the underlying mechanisms. Strategies that span preconception and that ensure the optimal maternal health during pregnancy are crucial to reduce the developmental origins of disease in the future. Maintaining a healthy body weight during adolescence could mitigate the negative effects of disadvantageous intrauterine environment or birth weight, and healthcare providers and families should take interventions and strategies targeting obesity and MetS in teenagers. This will also reduce the social and economic burden on society.

## Figures and Tables

**Table 1:** Definitions of metabolic syndrome in children *.

	Cook et al. [12]	De Ferranti et al. [13]	Weiss et al. [14]	Cruz et al. [15]
Abdominal obesity	WC > 90th p	WC > 75 p	BMI ≥ 2 SDS	WC ≥ 90th p (NHANES III)
HDL-c	≤40 mg/dL	<50 mg/dL	≤5 th p (NGHS)	≤10th p (NHANES III)
Triglycerides	≥110 mg/dL	≥100 mg/dL	≥95th p (NGHS)	≥90th p (NHANES III)
Blood pressure	≥90th p	>90 p	≥95th p	≥90th p
Hyperglycemia	FPG ≥ 110 mg/dL	FPG ≥ 110 mg/dL	GI (ADA criteria) ^#^	GI (ADA criteria)
MetS definition	≥3 criteria	≥3 criteria	≥3 criteria	≥3 criteria

ADA: American Diabetes Association; BMI: body mass index; FPG: fasting plasma glucose; GI: glucose intolerance; HDL: high-density lipoprotein; MetS: metabolic syndrome, NGHS: National Heart, Lung, and Blood Institute Growth and Health Study; NHANES III: Third National Health and Nutrition Examination Survey; p: percentile; SDS: standard deviation score; WC: waist circumference. * Criteria should be evaluated according to age and sex-specific reference values. ^#^ 2-h glucose ≥ 140 mg/dL.

**Table 2:** The International Diabetes Federation definition of metabolic syndrome in children and adolescents [16].

Age * (Years)	Abdominal Obesity (WC)	HDL-c	Triglycerides	Blood Pressure	FPG
6–<10	≥90th p	-	-	-	-
10–<16	≥90th p or adult cut-off	<40 mg/dL	≥150 mg/dL	SBP ≥ 130 or DBP ≥ 85 mmHg	≥100 mg/dL or known T2DM
16+	≥94 cm for males and >80 cm for females **	<40 mg/dL in males and < 50 mg/dL in females or treatment for low HDL	≥150 mg/dL or specific treatment for high TG	SBP ≥ 130 or DBP ≥ 85 mmHg or receiving hypertension treatment	≥100 mg/dL or known T2DM

DBP: diastolic blood pressure; FPG: fasting plasma glucose; HDL: high-density lipoprotein; p: percentile; SBP: systolic blood pressure; T2DM; type 2 diabetes mellitus, TG: triglycerides; WC: waist circumference. * MetS is not typically identified in children under the age of 10, but they will be advised to focus on weight reduction. Additional testing is recommended for children with a family history of MetS, T2DM, dyslipidemia, cardiovascular disease, hypertension, or obesity. MetS can be diagnosed in individuals who are 10 years old or older. Abdominal obesity must be present along with at least two of the other components. For adolescents aged 16 years and older, the IDF adult criteria can be applied, whereas a modified form of these criteria will be utilized for individuals aged 10 to 16 years old. ** These values are for European individuals. For individuals of South and South-East Asian, Japanese, and ethnic South and Central American origin, the cut-offs should be ≥90 cm for males and ≥80 cm for females.

## Data Availability

No new data were created or analyzed in this study. Data sharing is not applicable to this article.
